# Exploring the suitability of the Clark and Wells (1995) model of social anxiety in autistic adults: The role of mental imagery and fear of negative evaluation

**DOI:** 10.1177/13623613251379945

**Published:** 2025-10-30

**Authors:** Jiedi Lei, Juliette Attwood, Ailsa Russell

**Affiliations:** 1 University of Oxford, UK; 2 Essex Partnership University NHS Trust, UK; 3 University of Bath, UK

**Keywords:** Autism, cognitive therapy, fear of negative evaluation, imagery, social anxiety

## Abstract

Around 50% of autistic adults meet diagnostic criteria for social anxiety disorder based on self-report questionnaires. Among non-autistic adults with social anxiety, distorted negative self-imagery in social situations stemming from fear of negative evaluation from observers can be corrected in cognitive therapy via video feedback. However, the role of social imagery in the maintenance of social anxiety has not been explored in autistic adults. This study examined in 62 autistic adults: (1) quality of social imagery elicited during social situations; (2) how image qualities were related to self-reported levels of social anxiety and fear of negative evaluation when accounting for co-occurring generalised anxiety. Many autistic adults reported social imagery from a field (i.e., looking through one’s own eyes) rather than observer perspective. Using response surface analysis (RSA), autistic adults wanted to escape from/avoid social imagery and found them more upsetting when social anxiety was greater than fear of negative evaluation from others. Social imagery may be linked to autistic adults’ somatic and sensory responses related to social anxiety rather than cognitive worries associated with fear of negative evaluation from others in social situations. Future studies can explore how qualitative differences in social imagery may influence maintenance of social anxiety and treatment efficacy in autistic and non-autistic adults over time.

**Lay abstract:**

Many autistic adults experience social anxiety, which can negatively impact on one’s quality of life and increase risk for developing other mental health difficulties if left untreated. Current treatment for social anxiety involves supporting individuals to identify their worries in social situations and explore how focusing on one’s worries about being judged by others might generate an unhelpful and inaccurate negative image of oneself in social situations. In treatment, individuals use video feedback to look for differences between how they think they might come across to others, versus how they actually come across to others in conversation. Correcting any overly negative and unhelpful images of oneself in social situations is a key step in treatment for social anxiety. To date, little is known about whether autistic adults also generate negative images of oneself in social situations, and whether these images are related to one’s worries about being judged by others. In this study, we interviewed 62 autistic adults and asked them to generate images about relaxed and social situations. Autistic adults found images generated about being in a social situation to be more upsetting and anxiety inducing, less controllable and wanted to escape from/avoid such images. Negative aspects of social images were more related to general feelings of social anxiety rather than specific worries about being perceived negatively by others. We propose that autistic adults may draw on bodily sensations and sensory experiences related to general distress or feelings of discomfort to generate images in social situations. This is different to non-autistic adults where images may be generated based on one’s belief of how others might negative perceive oneself in social situations. Understanding such differences and the role images play in social anxiety for autistic and non-autistic adults can help clinicians better adapt treatment for social anxiety to suit autistic adults’ needs.

Social anxiety disorder (SAD) is characterised by a severe and persistent fear of social or performance situations (American Psychiatric Association, 2013) and has a 12% lifetime prevalence in the general population (Kessler et al., 2005). Without treatment, SAD is a highly disabling condition that predicts other serious problems such as depression, substance misuse and increased risk of suicide (Kessler et al., 2005; Rapee & Spence, 2004). One of the leading models underpinning the development of cognitive therapy for social anxiety (CT-SAD) recommended by NICE guidelines in the United Kingdom is the Clark and Wells (1995) cognitive model of social anxiety (SA). In this empirically supported model, individuals who perceive social situations to be threatening due to fear of negative evaluation (FNE) from others experience self-focused attention that heighten bodily sensations of anxiety, which in turn exacerbates negative self-processing, negative self-imagery, and leads the individuals to behave in a way that tries to prevent one’s worst feared social outcomes from happening (i.e., safety-seeking behaviours) (Clark & Wells, 1995). During treatment, clients experiment with shifting attentional focus both internally (i.e., paying attention to one’s worries, behaviours and bodily sensations) and externally (i.e., fully participating in the conversation/activity and paying attention to other people’s responses, emotional expressions and behaviours), drop safety-seeking behaviours (defined as ‘advanced, elaborate and creative strategies that aim to eliminate social-evaluative threat in these circumstances social situations without physically removing oneself’ in Wong & Rapee, 2016, p. 95) and test out whether one’s worst feared outcomes or cognitive beliefs about one’s social performance are true and proportionately accurate.

One mechanism proposed by the model to maintain SA is that internal focus of attention on the self particularly bodily sensations of anxiety and social worries can lead to experiencing negative imagery of how one might come across to others. Such imagery can be excessively negative, vivid, and perceived from an observer perspective (i.e., from the perspective of an outsider looking at oneself and seeing what one might look like, as opposed to field perspective where the image is perceived from one’s own perspective to look outwards) and serve to maintain negative self-beliefs, further increasing self-focused attention and perpetuate safety-seeking behaviours (Chiu et al., 2022). Adults with SAD are significantly more likely to report experiencing negative observer-perspective mental images of themselves when anxious in a social situation in comparison with non-socially anxious controls (Hackmann et al., 1998). They are also more likely to take an observer-perspective when asked to imagine a past anxiety-provoking social situation (Wells et al., 1998). NICE guidelines state that individual CT-SAD based on Clark and Wells model should include the use of video feedback aimed at correcting distorted negative self-imagery (National Institute for Health and Care Excellence, 2013). It has been found that these intrusive images tend to be linked to adverse events that occurred around the time of the onset of the disorder (Hackmann et al., 2000), and using imagery rescripting techniques for images relating to past events has also been shown to be an effective adjunct to cognitive therapy (Wild et al., 2007).

Autism is a neurodevelopmental condition characterised by social communication difference and a restricted, repetitive pattern of behaviour, interests or activities (American Psychiatric Association, 2013). The co-occurrence of SAD in autistic^1^ individuals is particularly high, with one study of a non-treatment-seeking population reporting 50% of autistic adults met diagnostic criteria for SAD and scored significantly higher on three self-report measures of SA (Maddox & White, 2015). Although research using screening measures of autism and SAD in young adults found evidence that the conditions are distinct (Lei & Russell, 2021; White et al., 2012), it is important to acknowledge that autistic individuals who experience high levels of co-occurring generalised anxiety (Lai et al., 2019) might find social situations to be overwhelming and result in social avoidance (Bellini, 2006). Cumulative negative social experiences can also contribute to the development of elevated anxiety in social situations (Bellini, 2004, 2006).

Autistic adults recounted in one qualitative study how autistic traits can be negatively perceived by others and described more frequent experiences of social trauma (Wilson, 2024). In non-autistic individuals, greater relational peer victimisation (e.g., social exclusion, bullying and neglect) and overt victimisation (e.g., physical/verbal aggression) have been shown to be the strongest risk factor for developing SAD over time (Siegel et al., 2009; Storch et al., 2005). Socially traumatic events may also result in hyperarousal, social avoidance, intrusive re-experiencing of the negative social event and negative changes in mood and cognitions (Norton & Abbott, 2017). It may be possible that autistic individuals who are more likely to encounter social adversity can subsequently develop more negative social imagery, and such imagery may be related to FNE from others both about their autistic traits and processes related to SA.

A recent study that compared autistic adults to non-autistic adults found that SAD-related cognitions, safety behaviours and self-focused attention accounted for almost half of the variance in social fears across groups, although the addition of autism diagnosis and its interaction with safety behaviours also accounted for additional variance in SA reported by participants (Wilson & Gullon-Scott, 2024). The interaction between autism diagnosis and safety behaviours may be related to the construct overlap between masking behaviours used to hide their autistic traits, and safety-seeking behaviours such as trying to come across well (impression management) that maintains SAD over time (Lei, Leigh, et al., 2024). In respect of the key cognitive processes outlined in the cognitive model of SAD, a recent systematic review found evidence in studies of autistic people for a number of these, particularly FNE from others and safety and avoidance behaviours, although no quantitative studies to date have explored the role of social imagery specifically in relation to SAD in autistic individuals (Lei, Mason, et al., 2024).

Given the evidence that autistic people tend to think more in image form (Hare et al., 2015), and in realistic, photograph like, pictures from memory (Crespi et al., 2016), it is possible that higher use of mental imagery in autistic people may be an important contributory factor to the heightened prevalence of co-occurring SAD. To date, only one study has used an imagery interview technique to look at how autistic and non-autistic children with and without general anxiety difficulties may experience social versus non-social imagery (Ozsivadjian et al., 2017). The study found that although autistic children with high levels of general anxiety generated more spontaneous anxious imagery than non-autistic children with anxiety, characteristics such as imagery vividness, realism and controllability did not differ between the two groups (Ozsivadjian et al., 2017). In a qualitative interview study with six autistic adults, Spain et al. (2020) found that only two adults reported noticing visual imagery from observer perspective when feeling anxious in social situations, although all participants reported noticing a range of physiological symptoms (e.g., ‘stomach churning away . . . being sick . . . get headaches’, ‘increased heart rate, a bit shaky’) indicative of anxiety in social situations.

Investigating the significance of negative self-imagery in respect of autistic people’s experience of SA is important in furthering our understanding of the goodness of fit of CT-SAD underpinned by the Clark and Wells (1995) model. In this study, we investigated how autistic adults with varying degrees of SA experience imagery in social versus non-social imagery conditions. We also wanted to understand whether FNE from others may be a maintenance process that is specifically related to anxiety in social situations (i.e., SA), as opposed to feelings of anxiety more generally that may also be related to a combination of mechanisms proposed to maintain anxiety more generally in autistic people (e.g., intolerance of uncertainty, sensory differences and emotion dysregulation as outlined elsewhere by Boulter et al. (2014), Riedelbauch et al. (2024) and Rodgers et al. (2018)). Acknowledging that FNE from others may encompass perceived stigma stemming from reduced external acceptance from society, family and friends (Cage et al., 2018), we wanted to explore whether distress associated with social imagery may be more specifically related to FNE from others, or SA symptoms more generally, when controlling for generalised anxiety. Based on predictions from the Clark and Wells (1995) cognitive model of SA, we hypothesised:

H1. Using a within-subject design, imagery generated by autistic adults with SA will be more distressing in a social image condition compared with a relaxed image condition, and autistic adults will be more likely to adopt the observer perspective in social imagery compared with relaxed imagery.
H2. FNE will show greater positive association with SA compared with generalised anxiety symptom severity in autistic adults.
H3. Greater SA and FNE may be associated with more distressing social imagery in autistic adults (e.g., upsetting, wanting to escape from the image and anxiety elicited by the image).

## Method

### Participants

Participants were adults (⩾18 years) with a clinical diagnosis of autism recruited online through social media, charities and local support services. Participants were mostly aged 25–34 or 35–44, female, Caucasian, educated to master’s or college level, in paid full-time or part-time employment, and had received their formal diagnosis of autism as an adult (Table 1). Participants completed an online survey via Qualtrics and were asked to confirm their diagnosis via self-disclosure in response to the question ‘Do you have a formal diagnosis of autism/Asperger’s?’. Participants also provided information about if they received their formal diagnosis before or after their 18th birthday and were not asked to provide their specific diagnosis. Participants completed demographic questions about their age, gender identity, ethnicity, highest level of education completed and current employment status via multiple-choice questions. Participants provided written informed consent to take part in an imagery interview.

**Table 1.** Participant demographic variables (*N* = 62).

	Frequency	Percentage
Age (years)		
18–24	9	14.5
25–34	20	32.3
35–44	17	27.4
45–54	8	12.8
55–64	8	12.9
Gender identity		
Male	19	30.6
Female	35	56.5
Non-binary	8	12.9
Ethnicity (*n* = 55)		
White	50	90.9
Asian	3	5.5
Mixed race	2	3.6
Education attainment		
GCSEs	5	8.1
A levels	10	16.1
College	5	8.1
Apprenticeship/Diploma	4	6.4
Bachelor’s degree	17	27.4
Master’s degree or above	17	27.5
Prefer not to say	4	6.5
Employment		
Full time – paid	18	29.0
Part time – paid	13	21.0
Part time – voluntary	3	4.8
Unemployed – looking for work	2	3.2
Unemployed – not looking for work	7	11.3
Retired	1	1.6
Prefer not to say	4	6.5
Student	14	22.6
Place of autism diagnosis		
NHS clinic	47	75.8
Independent practitioner	10	16.1
Other – charity	2	3.2
Prefer not to say	3	4.8
Age of diagnosis		
Under 18 years	7	11.3
18 years or older	54	87.1
Unsure	1	1.6

### Procedure

Participants read information about the study and completed written informed consent, a demographics questionnaire and a series of standardised self-report measures via Qualtrics. Participants then completed the mental imagery interview via the telephone. All interviews were recorded using a Dictaphone and transcribed verbatim. The online survey and imagery interview were both independently piloted with two autistic people, once in person and once over the telephone.

### Community involvement statement

Two autistic adults were involved in the designing and piloting of the research methodology, including testing the feasibility and acceptability of implementing and completing the mental imagery interview to assess imagery in social and relaxed conditions.

### Measures

#### Autism Quotient

The Autism Quotient (AQ-10) is a measure of autistic traits, with a cut-off point of 6 to indicate good sensitivity (0.88) and specificity (0.91) in adults (Allison et al., 2012).

#### Social Phobia Inventory

The Social Phobia Inventory (SPIN) is a measure of SAD. Seventeen items are rated using a 5-point scale, and scores range from 0 to 68. Higher scores indicate higher SA, and a score of 19 and below indicates no SA. The measure has demonstrated good test–retest reliability and excellent internal consistency (α = 0.94) (Connor et al., 2000) and has been used with autistic young people (Lei, Leigh, et al., 2024; Wood et al., 2022) with good internal consistency (α = 0.92 for both studies).

#### Brief Fear of Negative Scale

The Brief Fear of Negative Scale (BFNE) measures the extent to which people experience FNE from others. Twelve items are rated using a 5-point scale, and scores range from 12 to 60 with higher scores indicating higher fear. The scale has demonstrated excellent internal consistency (α = 0.90) (Leary, 1983). This measure was included to control for FNE and has been used with an adult autistic sample before with excellent internal consistency (α = 0.94) (Maddox & White, 2015).

#### Spontaneous Use of Imagery Scale

The Spontaneous Use of Imagery Scale (SUIS) measures use of mental imagery in daily life, with 12 items where each describes a situation such as ‘When I think about visiting a relative, I almost always have a clear picture of him or her’., and respondents are asked to rate on a scale of 1 (never appropriate) to 5 (always completely appropriate) (Reisberg et al., 2003). Scores range from 12 to 60 with higher scores indicating higher use. The scale has demonstrated good reliability, convergent validity with measures of imagery ability and good internal consistency (α = 0.86) (Nelis et al., 2014; Reisberg et al., 2003). This measure has not been validated in an autistic sample.

#### Generalised Anxiety Disorder 7-item

The Generalised Anxiety Disorder 7-item (GAD-7) rates the severity of anxiety symptoms. Seven items are rated on a 4-point scale, and scores range from 0 to 21 with higher scores indicating higher severity. The measure is widely used in U.K. primary care mental health services and has demonstrated excellent internal consistency in the general population (α = 0.92) (Spitzer et al., 2006). The measure has not been validated in an autistic sample; however, it was chosen to control for generalised anxiety because it uses concrete language and is quick and simple to complete.

#### Mental imagery interview

This is a structured interview originally developed by Hackmann et al. (1998) and adapted for autistic children by Ozsivadjian et al. (2017). Participants are asked to think about and generate images in both relaxed and anxiety-provoking social situations, and to rate several imagery characteristics (e.g., vivid, controllable, realistic, upsetting, making them feel like they want to leave the image and how anxious they felt) using a 10-point scale. To elicit mental images, participants were asked:

Now close your eyes and bring to mind a mental image of a situation that makes you feel happy and relaxed/a social situation that makes you feel anxious. Try and make the image as vivid as possible, like in a film, and when you’re ready tell me what’s happening.

After the participant finished describing their image they were asked: ‘In the image are you looking out at the world from your eyes or looking at yourself as if you were watching yourself on TV?’ The participants were then asked: ‘Keep the image in your mind. I’m now going to ask you to rate your image on some different characteristics using a 10-point scale’. Participants then rated the extent to which they experienced the image as vivid, controllable, realistic, upsetting, making them feel like they want to leave the image and how anxious they felt on a 0–10 scale. For example: ‘How vivid is the image? Vivid means it’s very bright and you can see all the details. On a 0–10 scale with 0 meaning not vivid at all and 10 meaning extremely vivid, how vivid is the image?’ Interviews were terminated if a participant could not recall a mental image for the first relaxed scenario. Before the SA image, participants were, in addition, asked: ‘When you are in a social situation that makes you feel anxious do you experience any mental images?’ and ‘How often do you experience images when you are in a social situation that makes you anxious? There are four options: 1 is never, 2 is sometimes, 3 is often, and 4 is always?’ Participants were asked about frequency regardless of whether they reported experiencing mental images and could then revise their previous answer to ‘Yes’ if they could provide an example of a mental image. See Supplemental Materials for details about the measure and adaptation for this study.

### Ethical approval

All procedures comply with the ethical standards of the relevant national and institutional committees on human experimentation and with the Helsinki Declaration of 1975 (revised in 2008). All procedures involving human participants were approved by the University Ethics Committee.

### Data analysis plan

Data were analysed using IBM SPSS version 29. Data were checked for outliers, normality and homogeneity of variance, and bootstrapping was applied if necessary. To test H1, we conducted Friedman tests and Wilcoxon signed-rank tests, using Bonferroni to correct for multiple comparisons. Next, we conducted Pearson’s bivariate correlations to explore associations between SA, FNE, generalised anxiety, spontaneous use of imagery and imagery characteristics in the social condition including imagery perspectives, using Benjamini-Hochberg (Benjamini & Hochberg, 1995) to correct for mulitple comparisons (given number of comparisons, Benjamini–Hochberg offers a less conservative yet effective control for false discovery rate for this exploratory analysis). We also completed chi-square tests to explore differences in perspectives reported in imagery generated in (1) Relaxed Condition 1 versus social condition and (2) Relaxed Condition 2 versus social imagery, using Bonferroni to correct for multiple comparisons.

Finally, we conducted RSA to test Hypotheses 2 and 3. First, we explored to what extent assumed similarity in ratings of SA and generalised anxiety is associated with FNE, when controlling for autistic traits (H2). We included the following predictors: (1) self-report SA symptom severity (X), (2) self-report generalised anxiety symptom severity (Y), (3) the quadratic term of SA symptom severity (X^2^), (4) the interaction between SA and generalised anxiety symptom severity (X*Y) and (5) the quadratic term of generalised anxiety symptom severity (Y^2^). Next, we explored to what assumed similarity in ratings of SA and FNE are associated with imagery characteristics in the social condition, when controlling for generalised anxiety (H3). We included the following predictors: (1) self-report SA symptom severity (X), (2) self-report FNE (Y), (3) the quadratic term of SA symptom severity (X^2^), (4) the interaction between SA and FNE (X*Y) and (5) the quadratic term of FNE (Y^2^). All predictors were centred on the questionnaires’ midpoint. RSA has the advantage of modelling both *linear* and *curvilinear* relationships at different levels of both *matches* and *mismatches* between two predictors in relation to an outcome variable (Barranti et al., 2017). We report the lines of congruence and incongruence as outlined by Barranti et al. (2017) and also the four-step checklist from Humberg et al. (2019) to explore the congruence hypothesis for each analysis, through combining the lines of congruence, incongruence and first principal axis. For all RSA models, the congruence hypothesis states that the outcome variable is higher when the two predictor variables are matched to one another.

### Sample size and power

For repeated measures analysis of variance (ANOVA), an a priori power analysis using G*Power indicated that to have an 80% chance of finding a medium-sized effect (*f* = 0.25) would require 28 participants. For non-parametric tests, power calculations using G*Power indicated that to have an 80% chance of finding a medium-sized effect (*dz* = 0.5) would require 35 participants. For RSA, an a priori power analysis using G*Power indicated that to have 80% chance of finding a medium-sized change (*f*^2^ *=* 0.15) in *R*^2^ from a two main effects model to a polynomial model by adding in the interaction and two quadratic terms would require 68 participants.

## Results

### Participant characteristics and questionnaire reliability

Participant characteristics on the key outcome measures are shown in Table 2. The SPIN and BFNE both showed excellent internal consistency (α = 0.91; 0.94, respectively), and the GAD-7 and SUIS both showed good internal consistency (both α = 0.88). Table 2 shows the percentage of participants that exceeded suggested cut-offs for SA and generalised anxiety across SPIN, BFNE and GAD-7 and highlights that most participants experienced high levels of SA (83%–92%) and many (68%) experienced moderate to severe levels of generalised anxiety.

**Table 2.** Participant characteristics on core outcome variables (*N* = 62).

	*M* (*SD*)	Range	*n* (%) ⩾ cut-off^a^
Autism Quotient-10	8.5 (1.41)	5–10	58 (93.55)
Social Phobia Inventory	34.18 (14.21)	11–62	52 (83.87)
Brief Fear of Negative Evaluation	43.81 (11.39)	19–60	57 (91.94)
Generalised Anxiety Disorder-7	12.68 (5.57)	1–21	Mild: 10 (16.13) Moderate: 18 (29.03) Severe: 27 (43.55)
Spontaneous Use of Imagery Scale	35.76 (11.50)	14–60	Not applicable

a Autism-Quotient-10 cut-off ⩾ 6; Social Phobia Inventory cut-off ⩾ 19; Brief Fear of Negative Evaluation cut-off ⩾ 25; Generalised Anxiety-7 cut-offs for mild, moderate and severe anxiety are 5, 10 and 15, respectively.

**H1:** Imagery in social situations will be more distressing than in relaxed conditions.

When participants were asked if they experience any mental imagery in social situations that make them feel anxious, 39 (62.9%) answered ‘Yes’, 22 (35.5%) answered ‘No’, and only 1 (1.6%) participant answered that ‘they were unsure’. When asked about the frequency of experiencing social imagery, 19 (30.6%) answered they never experience imagery, 17 (27.4%) answered sometimes, 17 (27.4%) answered often, and 8 (12.9%) answered always. All participants were asked to generate an imagery when thinking of a social situation that evoked anxiety. Table 3(a) shows the ratings of image characteristics for each image condition. Bonferroni correction was applied so the significance level was *p* < .008. Friedman tests showed that there was a significant effect of image condition for ratings of upset, escape/avoidance, anxiety and controllability. Post hoc Wilcoxon signed-rank tests showed that upset, escape/avoidance, and anxiety were rated significantly higher in the SA image (hereafter social imagery) condition in comparison with both relaxed image (hereafter relaxed imagery) conditions (completed before and after the social imagery). Controllability was rated significantly lower in the social imagery in comparison with Relaxed Image 1 and 2 conditions. No differences were found in vividness and realism across image conditions and suggested that autistic adults attended to all images equitably in both social and relaxed conditions.

**Table 3.** (a) Image characteristics for the three image conditions for the whole sample (*N* = 62), Friedman tests and Wilcoxon signed-ranks tests.

	Relaxed image 1	Social anxiety image	Relaxed image 2	χ² (2)	*p*
Vividness	7.32 (2.27)^c^	7.58 (1.99)^c^	7.70 (2.37)^c^	2.16	.340
Control	6.99 (2.95)^a^	4.07 (2.89)^a/b^	6.50 (2.76)^b^	35.49	.000
Realism	5.93 (2.90)^c^	6.34 (2.87)^c^	6.83 (2.75)^c^	7.72	.021
Upset	0.31 (0.74)^a^	6.24 (2.47)^a/b^	0.30 (0.83)^b^	113.68	.000
Escape	1.13 (1.58)^a^	7.22 (2.65)^a/b^	0.94 (1.69)^b^	90.70	.000
Anxiety	1.19 (1.71)^a^	7.31 (2.18)^ab^	1.15 (1.73)^b^	101.27	.000

a/b = *p* < .008 and ^c^ = *p* > .008 (Bonferroni corrections for multiple comparisons), ^a^shows significiant differences between Social Anxiety Image vs. Realxed Image 1 condition, and ^b^shows significant differences bewteen Social Anxiety Image vs. Relaxed Image 2 condition

**Table:** (b) Image perspective for the three image conditions for the whole sample (*N* = 62).

	Relaxed image 1 (*n*, %)	Social anxiety image (*n*, %)	Relaxed image 2 (*n*, %)
Field (looking out)	39 (62.9)	34 (54.8)	41 (66.1)
Observer (looking at self)	15 (24.2)	18 (29)	14 (22.6)
Both/switching	8 (12.9)	10 (16.1)	7 (11.3)

Table 3(b) shows number of individuals who endorsed different image perspectives in each condition. Bonferroni correction was applied so the significance level was *p* < .017. To evaluate whether there were significant differences in perspectives taken during relaxed versus social imagery, using chi-square tests, we found no significant association between perspective taken in relaxed condition one versus social condition (X^2^ (4, *N* = 62) = 10.76, *p* = .029), nor between perspective taken in relaxed condition two versus social condition (X^2^ (4, *N* = 62) = 7.21, *p* = .125). Overall, our findings partially supported H1, as social imagery was perceived by autistic adults to be more upsetting, more anxiety provoking, more likely to want to escape from/avoid the image, and less controllable compared with relaxed imagery, but was not more likely to be perceived from an observer perspective.

**H2:** FNE will show greater positive association with SA compared with generalised anxiety symptom severity.

Table 4 shows bivariate Pearson’s correlation coefficients between SPIN, GAD-7, BFNE, SUIS and imagery characteristics in the SA condition. SA symptoms (SPIN) were significantly associated with generalised anxiety symptoms (GAD-7, *r* = 0.49) and FNE (BFNE, *r* = 0.56). Generalised anxiety was also significantly associated with FNE (*r* = 0.4). There was no significant difference between the correlation co-efficient of FNE with SA versus generalised anxiety (*z* = 0.45, *p* = .65). Spontaneous use of imagery was not associated with SA, generalised anxiety, nor FNE.

**Table 4.** Correlation matrix between key outcome variables in the social imagery condition (*N* = 62).

Measure	1	2	3	4	5
1. SPIN	–				
2. GAD-7	0.49*	–			
3. BFNE	0.56*	0.40*	–		
4. SUIS	0.08	0.10	−0.09	–	
5. Perspective	0.20	0.04	0.23	0.09	–
6. Imagery characteristics					
Vividness	0.07	0.17	0.11	0.49*	0.14
Controllability	−0.18	−0.24	−0.16	−0.07	−0.05
Realism	0.05	0.11	−0.03	0.64*	0.22
Upsetting	0.30	0.33*	0.07	0.49*	0.15
Escape	0.34*	0.15	0.05	0.25	0.10
Anxiety	0.37*	0.39*	0.14	0.32*	0.24

*Note.* SPIN = Social Phobia Inventory, GAD-7 = Generalised Anxiety Disorder 7, BFNE = Brief Fear of Negative Evaluation scale, SUIS = Spontaneous Use of Imagery Scale.

* *p* < .05, **p < .01 and ***p < .001 (adjusted by Benjamini–Hochberg corrections for multiple comparisons.)

**Figure 1.**
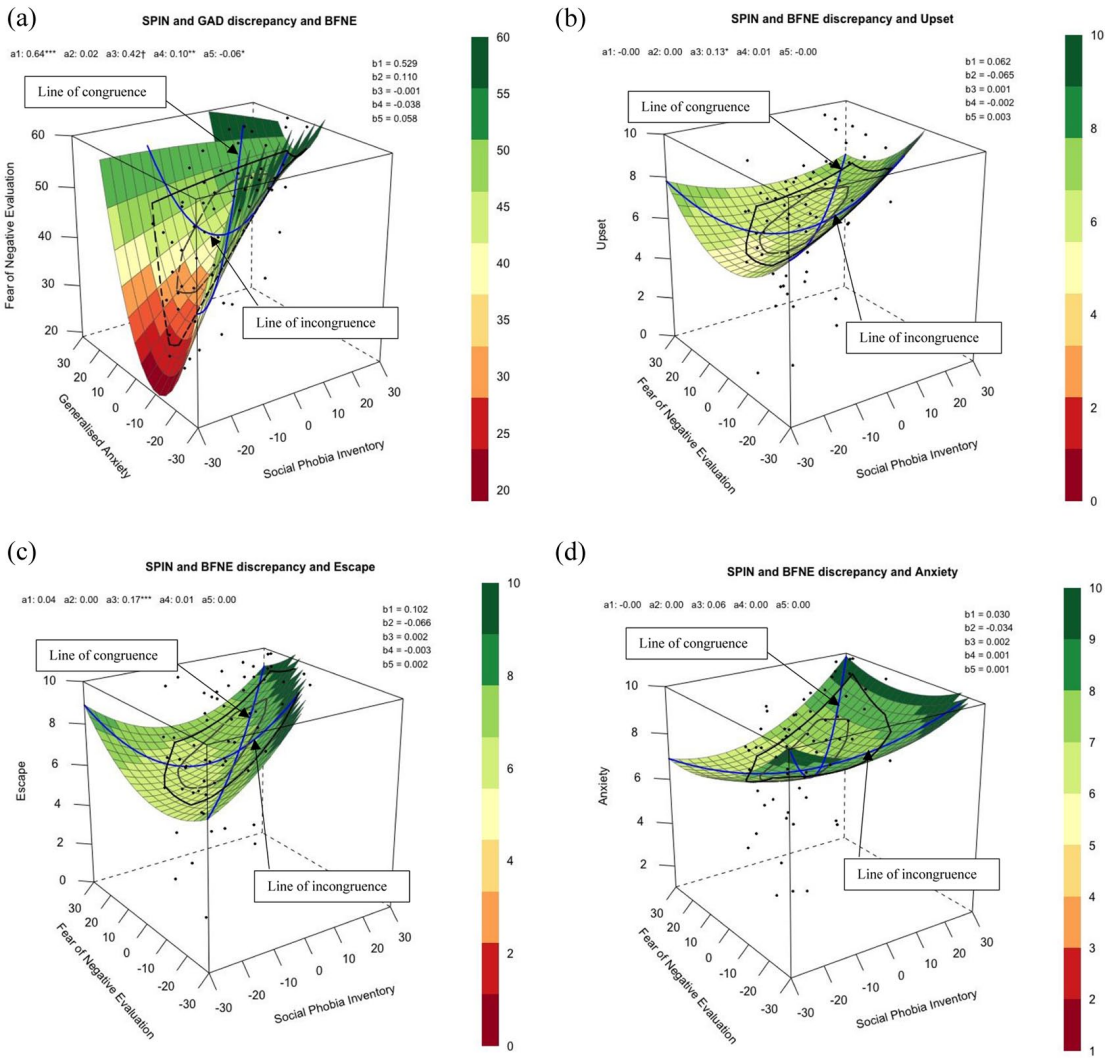
Response surface analysis for assumed similarity of (a) SA (predictor X) and generalised anxiety (predictor Y) when predicting fear of negative evaluation (outcome Z), controlling for autistic traits; and SA (predictor X) and fear of negative evaluation (predictor Y) when predicting imagery characteristics in the social condition such as (b) how upsetting the image was (outcome Z), (c) how much the participant wanted to escape from the image (outcome Z), and (d) how anxious the participant felt (outcome Z), when controlling for generalised anxiety symptom severity. *Note. X* and *Y* values of 0 reflect the midpoint of the scale, and legend shows the corresponding outcome *Z* values. The line of congruence (*a_1_* = slope and *a_2_* = curvature) shows cases where *X* and *Y* match perfectly (i.e., identical to each other), and line of incongruence (*a_3_* = slope and *a_4_* = curvature) where *X* are the opposites of *Y* values (both shown in blue). Bagplot (black lines superimposed on response surface) shows the prevalence of different combinations of *X* and *Y* predictors. More information on polynomial coefficients (*b* values; *b_0_* = intercept, *b_1_* *=* *X, b_2_* *=* *Y, b_3_* *=* *X*^2^, *b_4_* *=* X*Y, b_5_ *=* *Y*^2^) is reported in Table 5. *p-values are unadjusted for multiple comparisons.

To explore how self-reported ratings of social and generalised anxiety may relate to FNE by using RSA, Figure 1(a) shows how all combinations of participants’ self-reported SA (X) and generalised anxiety (Y) are associated with FNE (Z) when controlling for autistic traits (AQ). All polynomial coefficients are reported in Table 5. People expressed greater FNE when they reported dissimilar scores of SA and generalised anxiety, an association revealed by a positive curvature of the line of incongruence (*a_4_* = 0.1, *p* = .006). All mismatches equally contributed towards greater FNE, as it did not matter whether people reported to have greater SA than generalised anxiety or vice versa, an association revealed by the line of incongruence which was not statistically significant (*a_3_* = 0.42, *p* = .07). FNE was greater when participants perceived their GA and SA to be similarly high compared with being similarly low, an association revealed by the positive slope of the line of congruence (*a*_1_ = 0.64, *p* < .001). There was no curvilinear association along the line of congruence (*a*_2_ = 0.018, *p* = .493), meaning that FNE did not increase more sharply when SA and generalised anxiety were more similar to each other at extreme rather than midrange levels. When analysing the line of congruence, incongruence and first principal axis together, the RSA output did not support the congruence hypothesis. This means that FNE was not greater when self-reported ratings of SA and generalised anxiety were closer to one another, as the first principal axis significantly differed from the line of congruence (*p*_11_ confidence interval did not include 1).

**Table 5.** Response surface for assumed similarity of social anxiety (SPIN) and fear of negative evaluation (BFNE) in relation to characteristics of the imagery as reported by the sample in social condition (*N* = 62).(a) Estimated regression model.

	*B* (*SE*)	95% CI	β	*p*	*R*^2^ *(p*)
Model 1 (BFNE)					0.40 (<.001)
Covariates					
AQ-10	−1.31 (0.02)	−3.12, 0.51	−0.16	.16	
Predictors					δ*R*^2^ *(p*): 0.04 (<.001)
*b*_0_	42.88 (1.93)	39.09, 46.67	3.79	<.001**	
*b_1_* – SPIN	0.53 (0.07)	0.39, 0.67	0.66	<.001**	
*b_2_* – GAD	0.11 (0.2)	−0.28, 0.50	0.05	.58	
*b_3_* – SPIN^2^	−0.001 (0.006)	−0.01, 0.01	−0.01	.90	
*b_4_* – SPIN x GAD	−0.04 (0.012)	−0.06, −0.01	−0.29	.002**	
*b_5_* – GAD^2^	0.06 (0.03)	0.004, 0.11	0.19	.035	
Model 2 (upset)					0.17 (.10)
Covariates					
GAD-7	0.10 (0.06)	−0.0, 0.22	0.24	.084	
Predictors					δ*R*^2^ *(p*): 0.065 (.51)
*b*_0_	6.27 (0.67)	4.96, 7.58	2.56	<.001**	
*b_1_* – SPIN	0.06 (0.03)	0.004, 0.12	0.36	.036	
*b_2_* – BFNE	−0.06 (0.04)	−0.14, 0.007	−0.30	.076	
*b_3_* – SPIN^2^	0.001 (0.002)	−0.003, 0.005	0.08	.58	
*b_4_* – SPIN × BFNE	−0.002 (0.003)	−0.008, 0.003	−0.18	.42	
*b_5_* – BFNE^2^	−0.003 (0.003)	−0.003, 0.008	0.18	.39	
Model 3 (escape)					0.17 (.11)
Covariates					
GAD-7	−0.008 (0.073)	−0.151, 0.134	−0.017	.91	
Predictors					δ*R*^2^ *(p*): 0.14 (.11)
*b*_0_	7.20 (0.74)	5.75, 8.65	2.74	<.001**	
*b_1_* – SPIN	0.10 (0.03)	0.04, 0.17	0.55	.003*	
*b_2_* – BFNE	−0.07 (0.03)	−0.13, −0.007	−0.29	.029	
*b_3_* – SPIN^2^	0.002 (0.002)	−0.002, 0.006	0.16	.25	
*b_4_* – SPIN × BFNE	−0.003 (0.004)	−0.01, 0.004	−0.23	.37	
*b_5_* – BFNE^2^	0.002 (0.004)	−0.005, 0.009	0.13	.59	
Model 4 (anxiety)					0.24 (.015)
Covariates					
GAD-7	0.13 (0.04)	0.05, 0.20	0.32	.001**	
Predictors					δ*R*^2^ *(p*): 0.09 (.27)
*b*_0_	6.99 (0.56)	5.89, 8.08	3.22	<.001**	
*b_1_* – SPIN	0.03 (0.02)	−0.02, 0.08	0.20	.20	
*b_2_* – BFNE	−0.034 (0.03)	−0.10, 0.03	−0.18	.30	
*b_3_* – SPIN^2^	0.002 (0.001)	−0.001, 0.004	0.14	.29	
*b_4_* – SPIN × BFNE	0.001 (0.002)	−0.004, 0.005	0.05	.79	
*b_5_* – BFNE^2^	0.001 (0.002)	−0.004, 0.006	0.09	.64	

*Note.* Change scores are calculated from baseline model with covariates only. BFNE = Brief Fear of Negative Evaluation; GAD-7 = Generalised Anxiety Disorder-7; SPIN = Social Phobia Inventory.

* *p_adjusted_* < 0.05 and **p_adjusted_ < 0.01 (using Benjamini–Hochberg corrections).

**Table:** (b) Position of first principal axis and shape of surface along the lines.

RSA analysis	Position of first principal axis				Shape of surface along the lines								Final conclusion
					Line of congruence (LOC)				Line of incongruence (LOIC)				
	*p*_10_		*p*_11_		*a*_1_		*a*_2_		*a*_3_		*a*_4_		
	Est (*SE*)	95% CI	Est (S)	95% CI	Est (*SE*)	95% CI	Est (*SE*)	95% CI	Est (*SE*)	95% CI	Est (*SE*)	95% CI	
BFNE	143.95 (132.95)	−116.63, 404.52	−3.35* (1.55)	−6.38, −0.31	0.64*** (0.19)	0.26, 1.02	0.02 (0.03)	−0.03, 0.07	0.42 (0.23)	−0.03, 0.87	0.10** (0.03)	0.03, 0.16	No congru eff. (*p*_11_ CI excludes 1)
Upset	−62.53 (281.50)	−614.26, 489.21	−1.81 (1.84)	−5.42, 1.79	−0.003 (0.04)	−0.08, 0.07	0.001 (0.003)	−0.004, 0.007	0.13* (0.05)	0.03, 0.23	0.006 (0.007)	−0.007, 0.019	No congr eff. (*a_4_* not sig negative)
Escape	−32.40 (134.53)	−296.08, 231.27	−0.93 (0.92)	−2.73, 0.88	0.036 (0.043)	−0.05, 0.12	0.001 (0.003)	−0.004, 0.006	0.17*** (0.05)	0.08, 0.26	0.008 (0.008)	−0.009, 0.024	No congru eff. (*p*_11_ CI excludes 1)
Anxiety	25.91 (73.11)	−117.39, 169.20	0.52 (1.46)	−2.35, 3.39	−0.004 (0.03)	−0.06, 0.05	0.003 (0.002)	−0.001, 0.007	0.06 (0.05)	−0.03, 0.16	0.002 (0.005)	−0.009, 0.013	No congr eff. (*a_4_* not sig negative)

*Note*. Congr eff. = Congruence effect.

* *p* < .05, ** *p* < .01 and *** *p* < .001.

**H3:** Greater SA and FNE may be associated with more distressing social imagery.

As shown in Table 4, greater SA was associated with greater perceived feelings of wanting to escape (*r* = 0.34) and experiencing anxiety (*r* = 0.37) during the social imagery. Generalised anxiety was also significantly associated with finding the social imagery upsetting (*r* = 0.33) and anxiety provoking (*r* = 0.39). Greater spontaneous use of imagery was associated with a perception of more vivid (*r* = 0.49) and realness (*r* = 0.64) of social imagery, as well as finding the imagery more upsetting (*r* = 0.49) and more anxiety provoking (*r* = 0.32). FNE and perspective of imagery (e.g., field, observer and switching perspectives) were not associated with any imagery characteristics in the social condition.

To explore how distressing characteristics of social imagery may be related to both FNE and broader SA using RSA, Figure 1(b) to (d) shows how predictors SA (X) and FNE (Y) are associated with how upsetting, wanting to escape and anxiety-provoking social imageries were when feeling anxious (Z) when controlling for generalised anxiety. All polynomial coefficients are shown in Table 5.

In Model 2 (imagery characteristic: upset), analysing the line of congruence, how upset one felt about the social imagery generated was not associated with similarity in self-reported symptoms of SA and FNE at high or low levels (*a*_1_ = −0.003, *p* = .94), nor at midrange or extreme levels (*a*_2_ = 0.001, *p* = .67). Analysing the line of incongruence, imagery generated in social situations was rated as more upsetting when SA is higher than FNE (*a_3_* = 0.13, *p* = .02), but not associated with whether SA and FNE were similar to or different from one another (*a_4_* = 0.006, *p* = .37). When analysing the line of congruence, incongruence and first principal axis together, the RSA output did not support the congruence hypothesis. This means that imagery generated in social situation was not perceived to be more upsetting when self-reported ratings of SA and FNE were closer to one another (as curvature of line of incongruence shown by *a_4_* was not significantly negative).

In Model 3 (imagery characteristic: escape), analysing the line of congruence, how much one wanted to escape from the social imagery generated was not associated with similarities in self-reported symptoms of SA and FNE at high or low levels (*a*_1_ = 0.036, *p* = .40), nor at midrange or extreme levels (*a*_2_ = 0.001, *p* = .73). Analysing the line of incongruence, participants reported wanting to escape more from the social imagery when SA is higher than FNE (*a_3_* = 0.17, *p* < .001), but not associated with whether SA and FNE were similar to or different from one another (*a_4_* = 0.008, *p* = .37). When analysing the line of congruence, incongruence and first principal axis together, the RSA output did not support the congruence hypothesis. This means that participants did not want to escape more from imagery generated in social situation when self-reported ratings of SA and FNE were closer to one another, as the first principal axis significantly differed from the line of congruence (*p*_11_ confidence interval did not include 1).

In Model 4 (imagery characteristic: anxiety), analysing the line of congruence, how anxious one felt in the social imagery generated was not associated with similarities in self-reported symptoms of SA and FNE at high or low levels (*a*_1_ = −0.004 *p* = .89), nor at midrange or extreme levels (*a*_2_ = 0.003, *p* = .10). Analysing the line of incongruence, how anxious one felt in the social imagery was not associated with differences between SA and FNE (*a_3_* = 0.06, *p* = .19), and not associated with whether SA and FNE were similar to or different from one another (*a_4_* = 0.002, *p* = .71). When analysing the line of congruence, incongruence and first principal axis together, the RSA output did not support the congruence hypothesis. This means that participants did not report feeling more anxious in the imagery generated in social situation when self-reported ratings of SA and FNE were closer to one another (as curvature of line of incongruence shown by *a_4_* was not significantly negative).

## Discussion

This study investigated the association between different social imagery characteristics generated by autistic adults in relation to symptoms of SA, FNE and generalised anxiety. It was feasible to conduct an imagery interview with a relatively large number of autistic adults recruited online who reported high levels of SA symptoms on average. The majority reported experiencing negative mental imagery in social situations at least ‘sometimes’ which is consistent with research with neurotypical adults (Hackmann et al., 1998) (63% and 62%, respectively). Autistic adults reported finding social imagery to be more upsetting, more likely to elicit feelings of wanting to escape/avoid such imagery, more anxiety provoking and less controllable compared with relaxed imagery. This finding is in line with a previous study that found both autistic children with high and low anxiety experienced intrusive mental images (Ozsivadjian et al., 2017).

Autistic adults did not report significant changes from field to observer perspective when comparing relaxed imagery to social imagery. This is in line with a qualitative study with six autistic adults where only two reported experiencing social imagery form an observer-perspective (Spain et al., 2020), and in contrast to previous studies in non-autistic adolescents (Chiu et al., 2022) and adults (Dobinson et al., 2020) where greater SA was associated with observer-perspective social imagery. This finding suggests that social imagery generated by autistic adults may not suggest greater self-focused attention and shifting of perspectives from field to observer stance as suggested by the Clark and Wells (1995) model. Furthermore, social imagery characteristics were not associated with FNE, suggesting that autistic adults who experience SA may not be typically generating an image of how they worry others might perceive them in social situations. Instead, autistic adults’ self-reported levels of social and generalised anxiety were both associated with distressing characteristics associated with social imagery, which may suggest that they were relying more on internal bodily sensations and discomfort associated with high anxiety more generally when generating social imagery. The main difference being that only SA, not generalised anxiety, was associated with autistic adults reporting wanting to escape from/avoid social imagery. This suggests that social imagery generated by autistic people may be less driven by the fear that people will see they are anxious and make judgements, but more driven by the fear that they will be anxious and be unable to cope with the social situation and thus needing to escape.

The preference for visual imagery processing without taking observer-perspective may also be associated with general cognitive information processing preference for autistic individuals, especially regarding the construct overlap between theory of mind and social cognition differences alongside visual perspective taking (Pearson et al., 2013). Visual perspective taking can be differentiated in two levels, the first relating to identifying what a person can and can’t see from different perspectives, and the second relating to understanding that two different people viewing the same object/scenery do not necessarily see it in the same way, and the latter may be particularly influenced by theory of mind and being able to process social cognitive information through multiple perspectives (Pearson et al., 2013). Therefore, it may be the case that more autistic adults can generate an imagery of themselves by drawing on their own bodily sensations of discomfort associated with anxiety but may not always imagine how their discomfort may be perceived by others through an observer perspective. The current finding is in contrast to previous research in non-autistic individuals with social-evaluative concerns, where taking an observer perspective is seen as an example of ‘self-focused attention’ within the cognitive model of SA (Clark & Wells, 1995). Cognitive therapy for SAD focuses on supporting socially anxious individuals to focus their attention externally, which has been shown to lead to a significant shift in social imagery from an observer to field perspective, and greater reductions in SA in non-autistic individuals compared with exposure alone (Wells et al., 1998). This study suggests that for autistic adults, perspective of social imagery may be less important in the maintenance of SA, compared with how anxiety provoking, upsetting and how much the individual wants to escape from and avoid that social imagery because of how it feels rather than how they might be perceived by others.

In this study, FNE was significantly associated with both generalised and SA, and the magnitude of the two associations did not significantly differ from each other. In relation to social imagery, greater SA and generalised anxiety were both associated with negative aspects of social imagery reported by autistic adults, although FNE was not. When controlling for generalised anxiety, autistic adults found social imagery to be more upsetting and wanted to escape/avoid them when SA was greater than FNE. Anxiety ratings for social imagery were not significantly associated with either SA or FNE when controlling for generalised anxiety, suggesting general anxiety, rather than SA per se, may be associated with autistic adults finding social imagery more anxiety provoking. The relationship between negative aspects of social imagery, SA and generalised anxiety may resonate with qualitative reports from autistic adults that describe their anxiety to be ‘trauma-based’, and that such trauma may be enacted in the moment as a ‘conditioned response’ in anxiety-provoking social situations as the body responds in a fight or flight fashion (Wilson, 2024). Rather than a ‘fear’ of negative evaluation from others that suggests that one’s social worries are not always supported by actual evidence, autistic adults have reported that past negative social experiences can be described as relentless (Wilson, 2024). Although not always deliberately hurtful, such negative social experiences can stem from discrimination tied to their neurodivergence rather than anxiety per se (Wilson, 2024). It may be that social imagery elicited in this study triggered automatic negative emotions based less on social cognitive worries such as how one might come across to others, but rather reflect intrusive re-experiencing of actual past social trauma that may activate avoidance, negative interpretations of the social event and low mood, as well as hyperarousal (Norton & Abbott, 2017).

### Clinical implications

The current findings suggest that it would be feasible for clinicians working with autistic adults with co-occurring SAD to include an assessment of mental imagery in social situations. This is in line with NICE guidance for working with neurotypical adults (National Institute for Health and Care Excellence, 2013). It is recommended that clinical interview is used to gather information about mental imagery and that a clear definition of what is meant by the term ‘mental imagery’ should be provided (Hales et al., 2015). Participants in this study were given a very clear description of what was meant by the term ‘mental imagery’ and given a concrete example if needed. Although this may not guarantee that all autistic adults were able to engage in mental imagery, it is of note that some participants initially answered ‘No’ to the question ‘When you are in a social situation which makes you feel anxious, do you ever experience any mental images?’ but then rated a frequency of ‘Sometimes’ and changed their answer to ‘Yes’ before providing an example. This suggests that it may be necessary for clinicians to ask more than once and allow autistic clients extra time when exploring mental imagery.

However, the current findings also highlight the complexity and nuance relating to the experience of social imagery for autistic adults, as the role of social imagery and related mechanism in the maintenance of SA may be different compared with non-autistic adults as suggested in the Clark and Wells (1995) cognitive model of SA. Negative valence associated with the imagery may not be perceived from an observer perspective, but rather reflect on autistic adults’ perception of themselves based on internal information. This suggests that scaffolding questions about social imagery when formulating SA in autistic adults may benefit from having more prompts around bodily sensations related to anxiety, and physical discomfort or sensory differences that they notice in the moment. It should be noted that some of these internal bodily cues may not be visible from an observer perspective, while eliciting significant distress to the autistic individual in the moment. This contrasts with routine practice where clinicians may ask clients with SA to imagine how they may see themselves as if observed by someone else (observer perspective), or what instructions they might wish to give to an artist or actress to paint or act out how they feel in the moment. Such standardised prompts may rely on the autistic individual to use perspective taking and look for visible signs of anxiety from an outsider’s perspective that may be less easy to understand and complete. Our results suggest that autistic adults can engage in generating and reporting on social imagery without difficulty, though not always from observer perspective. Clinicians using video feedback to help clients ‘update’ their belief on how anxious they look to others may not find the same mechanism of change to work for autistic clients, as their social imagery might be more related to how anxiety feels in the body in the moment, rather than cognitive worries of how one might come across to others in the social situation. Instead, exploring with autistic clients how they understand this social imagery and what meaning it conveys about themselves and their past and present social experiences may be more useful.

Although this study showed that SA, rather than FNE, was more significantly associated with negative aspects of social imagery in autistic adults, how imagery techniques may be usefully applied with autistic adults were not explored in the context of cognitive therapy for SAD. One systematic review found only four single case studies of using CBT to treat SAD in autistic individuals, all of which consisted of psychoeducation, developing a hierarchy of anxiety-provoking situation and exposure tasks, and none included imagery work (Spain et al., 2017, 2018). None of the case studies provided a rationale for why imagery work was not included, although the reviewers hypothesised that imagery work may be ‘too complex to understand, or may not be required’ for autistic people (Spain et al., 2017). Furthermore, a qualitative study with autistic adults suggested that while autistic adults could relate to some of the key mechanisms outlined by the Clark and Wells (1995) cognitive model of SA when conceptualising their experiences of SA and that the model could be used as a helpful starting point for understanding individual experiences, more nuanced adaptations are needed to account for how individual’s social communication differences and other non-social factors may influence social interactions, and the elevated risk for autistic adults to encounter traumatic social experiences (Wilson, 2024). Recommendations for adapted CBT for autistic individuals emphasise behavioural rather than cognitive change methods, however, if mental imagery can be subject to modification or rescripting, it may prove to be a useful tool for change at the cognitive level via visual as opposed to verbal means. Such imagery may also be a useful way to gain insight into physiological markers of anxiety for autistic individuals in social situations.

Finally, the differentiation that only SA, and not generalised anxiety, was associated with wanting to escape from/avoid social imagery also has implications for cognitive therapy for SAD in autism. Our findings suggest that perhaps updating one’s own imagery of how anxious might look based on video feedback may be less effective than working together with autistic adults to talk about how to manage anxiety in social situations and develop alternative coping strategies other than avoidance or masking. Clinicians may also need to hold in mind that such imagery may also recall past traumatic social memories that need to be met with understanding and validation by the clinician and may not reflect a FNE from others that is disproportional to the actual lived experiences of the autistic individual. It may also be possible that persistent negative social encounters have led to increased negative bias where autistic adults are more likely to assume that others will not like them compared with non-autistic adults (Gurbuz et al., 2024). Rather than drawing on video feedback to correct observer-perspective images as stated in the recommended protocol for SAD, careful assessment and collaborative formulation may lead clinician and client to consider making sense of past bullying and victimisation, with the purpose of imagery rescripting to update distressing social memories from the past, and to reduce the negative emotions elicited by socially traumatic memories (Wild et al., 2007).

A recent systematic review (Quinton et al., 2024) that explored the assessment and treatment of post-traumatic stress disorder in autistic people found that clinicians working with autistic individuals may often overlook trauma-related symptoms in response to events experienced by autistic people as traumas, which often fall beyond traditional definitions of traumatic events. The review highlighted that there is a gap in empirical literature that examined the emergence of complex PTSD following repeated exposures to traumatic circumstances such as negative social encounters in autistic people across the lifespan. The review also highlighted that interventions developed by non-autistic people which are designed to change behavioural repertoires of autistic children in line with neurotypical norms can give rise to post-traumatic stress and adverse effects. In addition, a recent empirical study also found that autistic adults are more likely to expect to be liked less by others (have a greater expectation of social rejection) compared with non-autistic adults (Gurbuz et al., 2024), a finding that is not so surprising when only 7% of autistic people feel accepted by society as an autistic person (Cage et al., 2018). Such findings suggest that a large part of social rejection sensitivity and FNE from others may be linked to how autistic traits and autism identity are perceived and responded to by others that may contribute to and interact with experiences of SA. The need for clinicians to understand SA through the lens of their autistic client and account for the impact of past negative experiences and social trauma has also been highlighted, as qualitative accounts from autistic adults view their SA as ‘trauma-based’, and their experienced SA is not disproportionate when considering their sociocultural context (Wilson, 2024; Wilson & Gullon-Scott, 2024). Given that this study found social imagery may not be uniquely associated with FAE nor the adoption of observer perspective when experiencing SA in autistic adults, clinicians need to carefully explore the information used to generate social imagery, how such bodily experiences may relate to past social experiences, and make informed decision together with the client around how best to experiment with behaviour change in a way that encourages more helpful coping skills with SA (Wilson, 2024). A proposed model to account for autism related characteristics and their impact on different SA maintenance processes outlined in the Clark and Wells (1995) model has also been proposed in a recent systematic review (Lei, Mason, et al., 2024), further emphasising the importance for clinicians to carefully consider the interaction between autism traits, social experiences and SA symptoms to inform treatment adaptation.

### Strengths, limitations and future directions

This is the first study to investigate the role of mental imagery in SA in autistic adults. The study also benefitted from the involvement of two autistic people in designing and piloting the methodology. It demonstrates that an imagery interview can be successfully used with this population. There are also several limitations in terms of design and conduct of the study. First, this study did not have a non-autistic group that had similar levels of SA symptoms, which would have enabled conclusions about similarities and differences in the relationship between SA symptoms and social imagery characteristics in the context of autistic traits to be drawn with greater confidence. This study can only conjecture based on past qualitative studies with autistic adults how their lived social experiences may have contributed towards the patterns of results observed. The SA scores in the current sample were very high compared with previous research which has found the rate of co-occurring SAD in autism to be 50% (Maddox & White, 2015). It may be that previous research involved telephone screening prior to data collection that may have stopped those with very high levels of SA from participating, and thus, the rates of elevated SA symptoms in autistic adults may be better reflected by the current sample. It may also be that this study was advertised as investigating mental imagery and social confidence, and, therefore, attracted individuals with an interest in SA.

Participants in this study also self-reported when and where their autism diagnosis was given, and no structured diagnostic tools were used to confirm or validate autism diagnosis. The use of screening tool recommended by U.K. clinical guidance does add some validity. More than two thirds of the participants interviewed reported receiving the diagnosis via a National Health Service (NHS) U.K. clinic. Most participants received a diagnosis in adulthood indicating they may not be representative of those who received their diagnosis in childhood. Most of the participants were also female, and their social experiences might have also been different due to cultural expectations that may or may not be related to differences in use of masking to hide their autistic traits and social communication differences (Alaghband-rad et al., 2023) and, therefore, may limit the generalisability of findings to all autistic people. We did not ask participants to report on their past experiences of negative social encounters, including victimisation such as bullying from peers as well as acts of discrimination they may have experienced. Therefore, although this study contextualises findings by relating to autistic adults’ qualitative account of the impact of social trauma on SA from other research (Wilson, 2024; Wilson & Gullon-Scott, 2024), it is unclear the extent to which participants in this study experienced similar social encounters to the same degree, and future research can benefit from incorporating questions about one’s social experiences into social imagery interview to understand how such imagery may relate to one’s own lived experiences. Finally, we also did not ask participants to share information about their co-occurring mental and physical health conditions and, therefore, are unable to specify whether there may be between-group differences in the experience of imagery and SA when looking at overall mental and physical health symptoms. We tried to account for potential impact of generalised anxiety on the associations between imagery and SA when conducting RSA, though highlight that future studies may wish to both have clear comparison groups that have clinically diagnosed co-occurring SA and/or depression in the absence of other co-occurring mental health difficulties, as well as collecting general information about mental and physical health to further help interpret, contextualise and assess the generalisability of the current findings to both clinical and non-clinical populations.

## Conclusion

This study suggests that autistic adults can generate social imagery which can be distressing when experiencing SA, although such imagery may not necessarily be perceived from an observer or onlooker perspective, nor related to FNE from others. Future studies that explore how social imagery may be linked to autistic individuals’ somatic and sensory responses in relation to SA, as well as how such imagery may be related to past traumatic social experiences may offer valuable insight into factors that maintain social avoidance in autistic adults.

## Supplemental Material

sj-docx-1-aut-10.1177_13623613251379945 – Supplemental material for Exploring the suitability of the Clark and Wells (1995) model of social anxiety in autistic adults: The role of mental imagery and fear of negative evaluation Supplemental material, sj-docx-1-aut-10.1177_13623613251379945 for Exploring the suitability of the Clark and Wells (1995) model of social anxiety in autistic adults: The role of mental imagery and fear of negative evaluation by Jiedi Lei, Juliette Attwood and Ailsa Russell in Autism

## Notes

1. In this article, we will use identity first language as it has been shown to be preferred by members of the autistic and autism community by Kenny et al. (2016).
