# Supplementary material for: Exploring the suitability of the Clark and Wells (1995) model of social anxiety in autistic adults: The role of mental imagery and fear of negative evaluation
Source: Autism. 2025 Oct 30;30(1):228–44. doi: 10.1177/13623613251379945 (PMC12717292; doi:10.1177/13623613251379945)
Supplement: sj-docx-1-aut-10.1177_13623613251379945 – Supplemental material for Exploring the suitability of the Clark and Wells (1995) model of social anxiety in autistic adults: The role of mental imagery and fear of negative evaluation [file sj-docx-1-aut-10.1177_13623613251379945.docx]

**Supplementary Materials**

*Information about measures Mental Imagery Interview*

This is a structured interview originally developed by Hackmann et al. (1998) and adapted for autistic children by (Ozsivadjian et al., 2017). In the original interview, mental images are defined and participants rate how frequently they experience images before or during anxiety provoking social situations using a 4-point scale. Participants then think about a time recently when they felt anxious in a social situation and bring to mind an image of the situation. Participants then rate what perspective they are seeing the image from, the extent to which the image seems distorted, and how clearly they see the image. The adapted version included an initial relaxed scenario to serve as a control and practice condition, a suggested as well as self-generated anxious condition, and questions exploring emotional valence and whether the self-generated image was related to an actual event. Only a self-generated condition was necessary for the purposes of this study and the practice condition was retained as it was felt that this would help ensure that the participants fully understood the task. In the version used in the present study, participants imagine a relaxed situation, then a situation in which they feel socially anxious, and then a relaxed situation again. All participants were given the same verbal instructions using an interview script. Participants were not asked if the social anxiety image seemed distorted or was related to an actual event because this information was not required. It was also felt that the telephone methodology meant that potential distress triggered by asking the latter could not be adequately managed.
